# Intraoperative Monitoring of the Facial Nerve during Microvascular Decompression for Hemifacial Spasm

**DOI:** 10.3390/life13071616

**Published:** 2023-07-24

**Authors:** Chiman Jeon, Na Young Jung, Minsoo Kim, Kwan Park

**Affiliations:** 1 Department of Neurosurgery, Korea University Ansan Hospital, Ansan 15355, Republic of Korea; caudate@daum.net; 2 Department of Neurosurgery, Ulsan University Hospital, University of Ulsan College of Medicine, Ulsan 44033, Republic of Korea; ehdskswldl@uuh.ulsan.kr; 3 Department of Neurosurgery, Gangneung Asan Hospital, Gangneung 25440, Republic of Korea; 4 Department of Neurosurgery, Konkuk University Medical Center, Seoul 05030, Republic of Korea; kwanpark@skku.edu

**Keywords:** hemifacial spasm, microvascular decompression surgery, intraoperative neurophysiological monitoring, facial nerve

## Abstract

This review article discusses the clinical significance of intraoperative neurophysiological monitoring (IONM), provides recommendations for monitoring protocols, and considers the interpretation of results in microvascular decompression (MVD) for hemifacial spasm (HFS). The lateral spread response (LSR) is an important monitoring parameter during MVD. It helps to identify the responsible blood vessel and confirms its thorough decompression from the facial nerve. The disappearance of the LSR during surgery is associated with favorable clinical outcomes. Standard and revised monitoring protocols and the confirmation of LSR persistence and disappearance are also discussed. The blink reflex and other facial nerve monitoring modalities, such as free-running electromyography, facial motor evoked potentials, F-waves, and the Z-L response, are further considered.

## 1. Introduction

Hemifacial spasm (HFS) is a neurological disorder characterized by involuntary spasms of the muscles innervated by the facial nerves on the affected side of the face. Although the pathogenesis has not been clarified so far, axono-axonal ephaptic transmission caused by intracranial vascular compression of the facial nerve at the root exit zone of the pontomedullary junction is referred to as a peripheral mechanism [1,2], and hyperexcitability of the facial nucleus is considered the central mechanism [3,4].

Microvascular decompression (MVD) of the facial nerve is a treatment that can cure HFS. The purpose is to physically remove the previously described pathogenesis, and to detach the culprit vessel from the facial nerve. Many studies report a complete recovery rate of around 90% after surgery and hearing impairment and facial weakness as postoperative complications [5,6,7,8,9].

Intraoperative neurophysiological monitoring (IONM) represents a valuable tool in performing MVD for HFS [10,11,12,13,14]. First, through the IONM technique, it is possible to ensure the successful decompression of the culprit vessel from the facial nerve root exit zone. This is particularly valuable in cases where the culprit vessel is not evident in the surgical field. Second, the surgical manipulation of the cranial nerves, perforating vessels, and brainstem parenchyma may expose patients to a substantial risk of neurological injury. Vestibulocochlear nerve injury resulting in hearing impairment is the main concern in the MVD of the facial nerve for the treatment of HFS. From this perspective, IONM provides continuous functional feedback on the functional integrity of critical nerve function, as well as aiding in the achievement of the therapeutic goal, namely the detachment of the culprit vessel from the compressed facial nerve root entry zone.

This paper reviews the clinical significance of IONM for the facial nerve during MVD for HFS, suggests the most acceptable monitoring protocol and interpretation of the results, and summarizes recently published studies.

## 2. Microvascular Decompression

MVD is a widely accepted first-line surgical treatment for HFS that aims to detach the culprit vessel from the facial nerve [7]. No non-surgical treatment has shown satisfactory efficacy in the treatment of HFS, and the spontaneous resolution of HFS is very rare. However, MVD provides relief from spasm in approximately 90% of patients [5,6,8,9]. Patients whose symptoms are typical and whose MRI reveals a vessel compressing the facial nerve can be candidates for MVD. Secondary HFS caused by a cerebellopontine angle tumor or other space-occupying lesions that irritate the facial nerve also can be identified on MRI and treated with MVD.

The timing of MVD surgery is still under debate. Theoretically, MVD may not relieve symptoms in patients whose disease duration is long, because the facial nucleus could be degenerated. However, there is no prognostic difference between early and late MVD to date. Therefore, MVD is usually recommended when the patient experiences a severe, progressive facial spasm to the extent that the patient suffers difficulty in his/her social activity and decreased quality of life. Moreover, the patient needs to understand the aim, process, and risks of the surgery and have realistic expectations.

The notion that a normal vessel may cause cranial nerve disorders by compressing the respective nerve roots was introduced by Dandy and Gardner [15]. Janetta, in 1966, recognized that HFS was a cranial nerve hyperactive condition analogous to trigeminal neuralgia, and that HFS was caused by vascular compression of the facial nerve. Therefore, the first MVD for the treatment of HFS was performed by Janetta on a 41-year-old patient [16], who recovered well without complications; since then, the MVD technique has been developed on the basis of this original procedure.

A group of disorders, including HFS, trigeminal neuralgia, glossopharyngeal neuralgia, geniculate neuralgia, and hemimasticatory spasm, are referred to as neurovascular compression syndrome, which can be treated with MVD.

MVD for trigeminal neuralgia is quite analogous to that for HFS. The surgical approach for each disease is almost identical, as described by McLaughlin [17]. Unlike HFS, the operator’s approach uses the superolateral aspect of the cerebellum to gain access to the trigeminal nerve. During the procedure, the operator can encounter treatment-related morbidities, such as bleeding from the petrosal vein, venous infarction, sigmoid thrombosis, or hemorrhage from the cerebellum. Treatment outcomes of MVD for trigeminal neuralgia are no different from those of HFS. However, the recurrence rate is reported to be slightly higher for trigeminal neuralgia after MVD than for HFS [18,19].

Glossopharyngeal neuralgia is a relatively rare pain syndrome in the sensory distribution of the glossopharyngeal nerve. The pain is unilateral, sharp, and concentrated within the area of the sensory distribution of the nerve, including the angle of the jaw, ear, tonsillar fossa, and tongue base. Therefore, pharmaceutical management besides surgical intervention such as MVD is the first-line treatment. Selective nerve blockage also may be an option in patients who are refractory to medication; however, dysphagia, hoarseness, arrythmia, or syncope may occur following treatment. MVD has been established as an effective and safe treatment option for glossopharyngeal neuralgia [20,21,22].

Geniculate neuralgia (nervus intermedius neuralgia) is a pain syndrome in the ear, triggered by sensory or mechanical stimuli at the posterior wall of the auditory canal. Geniculate neuralgia is managed with medication, sectioning of the intermediate nerve (a sensory component of the facial nerve) [23], or MVD [24,25,26,27].

Authors have reported on the complications of microvascular decompression (MVD) for the treatment of hemifacial spasm (HFS) based on an analysis of 2040 patients [28]. In this study, immediate facial nerve palsy occurred in less than 1% of patients, while delayed facial palsy occurred in 7.3%. Serviceable hearing loss was observed in approximately 2.5% of cases, and non-serviceable hearing loss in 1.2%. Additionally, 5.3% of patients exhibited symptoms suggestive of CSF leaks, and 4.9% presented with middle ear effusion. Other reported complications included CSF rhinorrhea (0.6%), lower cranial nerve palsy (0.4%), dysgeusia (0.4%), meningitis (0.4%), wound problems requiring revision surgery (0.3%), vascular complications (0.3%), and sixth nerve palsy (0.1%).

Overall, MVD is considered a safe and effective treatment for HFS. The occurrence of major morbidities or permanent complications is rare. However, it is crucial for surgeons to remain vigilant and well informed about all potential complications, as well as remaining updated on advancements in surgical technology. By doing so, they can enhance the safety and effectiveness of MVD as a treatment option for HFS. Continuous education and awareness will contribute to better patient outcomes and a reduction in adverse events during the surgical procedure.

## 3. Intraoperative Neurophysiological Monitoring

IONM is essential in neurosurgical procedures and is gaining interest among both clinicians and researchers. Advances in technology over the past few decades have made IONM essential in various fields of medicine, such as orthopedics, otolaryngology, cardiac surgery, and even interventional radiology.

IONM was introduced in the form of direct cortical stimulation during an epilepsy surgery by Penfield [29,30]. Thereafter, electrocorticography, evoked potential, electromyography (EMG), and nerve conduction studies were applied during surgery [31]. Electroencephalography in cardiac surgery [32,33,34] and sensory and motor evoked potential monitoring in spinal cord surgery were introduced in the 1970s [35,36]. In the 1980s, motor evoked potentials were employed in evaluating corticospinal tract integrity, and somatosensory evoked potential and motor evoked potential monitoring became routine protocols in surgery. Later, the brainstem auditory evoked potential was introduced to monitor the whole auditory pathway and became essential, especially in surgery for infratentorial lesions.

Advancements in anesthetic techniques, as well as cooperation with anesthesiologists, are also key in IONM. Neural activity could become monitorable under a constant depth of anesthesia, especially due to the availability of total intravenous anesthesia, enabling surgical teams to obtain reliable responses [37,38,39,40].

However, IONM has been associated with several limitations. During somatosensory evoked potential monitoring, temporal summation can interfere with real-time interpretation, and distinguishing individual nerve root function can be challenging. Continuous monitoring with transcranial motor evoked potentials is difficult, and results can be misinterpreted due to neuromuscular blockage. Spontaneous EMG is sensitive to changes in body temperature, and false positive alarms may occur. Determining the threshold levels for individual cases is difficult in triggered EMG.

The current IONM setting has scope for improvement. Firstly, continuous monitoring requires dedicated, experienced personnel and cannot be carried out without specialized knowledge of specialized electrophysiology. Second, monitoring is a labor-intensive task that can be easily rendered inaccurate owing to fatigue and loss of concentration. Third, various clinical information, such as the patient’s individual clinical status, depth of anesthesia, and surgical situation, must be considered. However, no member of the surgical team can gather such information and interpret the IONM findings during surgery. Recently, initial research was reported on the use of artificial intelligence for automated interpretation, aiming to address these challenges [41].

In the field of MVD, it remains challenging to claim that standardized testing has been fully achieved. Modified protocols continue to be proposed. Despite these efforts, IONM still plays a supplementary role. In practice, many crucial decisions during surgery rely more on the operator’s experience than on IONM findings.

## 4. Lateral Spread Response (Abnormal Muscle Response)

**Figure 1:**
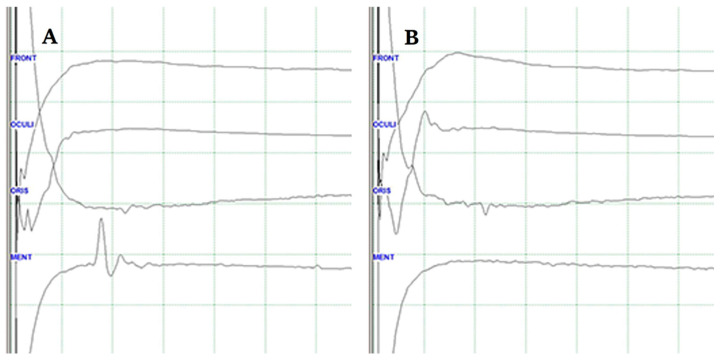
Lateral spread response (LSR) (**A**). The typical delayed electrical activity in the mentalis muscle following stimulation of the temporal branch of the facial nerve (**B**). LRS disappears during the procedure when the culprit vessel is removed from the facial nerve.

The lateral spread response (LSR) is used to identify the responsible blood vessel and ensure its thorough decompression from the root exit zone of the facial nerve during MVD for HFS. In over 95% of HFS patients, the LSR is recorded intraoperatively [42]. Once the culprit vessel is detached and adequately cushioned to prevent re-compression of the facial nerve, the LSR typically disappears within seconds to minutes (Figure 1) [43,44]. The intraoperative disappearance of the LSR has been associated with favorable clinical outcomes in the postoperative period, although its relationship with long-term outcomes remains uncertain [12,13,45,46]. However, recent studies indicate that the postoperative disappearance or reappearance of the LSR may serve as an indicator of long-term outcomes [42,47].

## 5. Standard Monitoring Protocol for the LSR

The LSR is acquired on the affected side of the face using bipolar pairs of needle electrodes for both stimulation and recording purposes. Traditionally, a bipolar electrode pair is positioned over the orbicularis oculi and mentalis muscles for recording. Another electrode pair for stimulation is inserted intradermally, approximately 1 cm apart, along the zygomatic branch of the facial nerve, which innervates the orbicularis oris muscle. While there may be some interpersonal variation, the typical location for the stimulation electrode is at the midpoint of an imaginary line between the tragus and the outer corner of the ipsilateral eye. In the standard methodology of LSR monitoring, it is recommended to achieve centripetal transmission of electrical stimulation towards the brainstem by placing the cathode in a proximal position relative to the anode.

## 6. Preoperative Mapping and Anesthetic Considerations

Authors have made modifications to their monitoring protocols based on experience with over 5000 cases of MVD. The standardization of the procedure relies on crucial considerations, such as anesthetic management, preoperative facial nerve mapping, and intraoperative monitoring protocols [48,49,50,51]. Sodium thiopental (5 mg/kg body weight) is used for intravenous anesthesia, followed by the use of a neuromuscular transmission module to establish the baseline twitch response at the ulnar nerve. During anesthesia maintenance, propofol (3–5.5 µg/mL) and remifentanil (1–4 ng/mL) are continuously infused to maintain the arterial blood pressure within a 20% range of its preoperative value [52,53]. IONM aims for consistency by targeting a bispectral index level of 40–60 and a train-of-four stimulation response at 50% twitch height relative to the baseline.

Preoperative mapping of the facial nerve branches plays a crucial role in facilitating the successful intraoperative monitoring of the LSR. Before surgery, the temporal branch of the facial nerve is identified and marked to enable the accurate stimulation of the facial nerve during the procedure. The site at which the amplitude of facial nerve excitation is maximal is determined by sweeping the skin with the cathode. These identified sites from the preoperative mapping serve as reference points for the placement of the stimulating cathode during surgery.

## 7. Revised Monitoring Protocol for the LSR (Figure 2)

The stimulating anode is placed anterior to the tragus, while the cathode is positioned on the previously identified temporal branch of the facial nerve, typically 3 cm superior to the anode. Recording electrodes are placed on specific muscles: the frontalis muscle (4 cm above the eyebrow), orbicularis oculi muscle (between the eyebrow and eyelid near the lateral palpebral commissure), orbicularis oris muscle (above the upper vermilion border between the nasolabial sulcus and philtrum), and mentalis muscle (below the lower vermilion border lateral to the mental protuberance). Authors have recently proposed an inversion of the relative location of the cathode and anode, which generates centrifugal stimulation outward from the brainstem, potentially improving the efficacy of LSR monitoring compared to the classical method [50]. The distance between the stimulating electrode and each recording electrode is kept constant.

**Figure 2:**
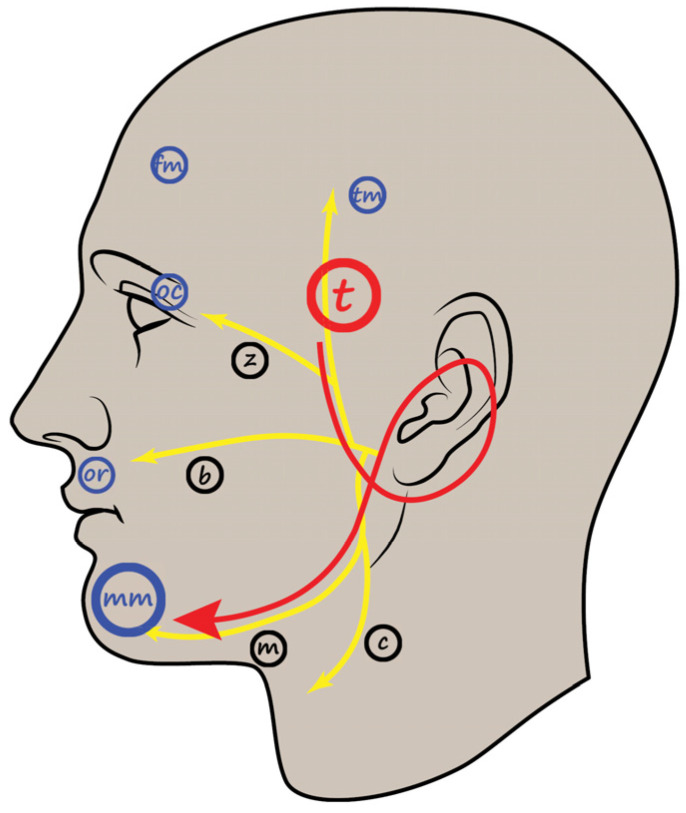
Schematic illustration of the LSR recording procedure. The recording electrodes for the mentalis muscle are inserted at the point shown in the figure. Yellow lines and black markings indicate facial nerve branches (t, temporal; z, zygomatic; b, buccal; m, mandibular; c, cervical branch). Blue markings indicate the facial muscles used in LSR recording (tm, temporal; fm, frontalis; oc; orbicularis oculi; or, orbicularis oris; mm, mentalis muscle). Red arrows and large markings indicate the direction of electrical stimulation, stimulating nerve branch, and recording muscle. LSR, lateral spread response. Adapted with permission from copyright holder, Prognostic Value of Lateral Spread Response Recorded 1 Month After Microvascular Decompression for the Treatment of Hemifacial Spasm, Neurosurgery, 91, 159–166, 2022 [42].

Initially, the stimulating parameters are set with an intensity of 5.0–25.0 mA and a duration of 0.3 ms. The threshold value for the stimulating intensity is determined by the point at which no further increase in the amplitude of the LSR is observed upon increasing the intensity by 1.0–2.0 mA. IONM is conducted from the time of anesthetic induction to the conclusion of the surgery, as unusual LSR findings may arise, such as early disappearance before decompression, persistence even after decompression, and reappearance after the prompt disappearance of the LSR [48].

## 8. Confirmation of a Positive LSR

The latency of the evoked response is used as a guide to confirm a positive LSR. The onset latency of the compound muscle action potential (CMAP) in each recording electrode of the four muscles should be less than 4 ms. On the other hand, the LSR typically exhibits longer latency, usually exceeding 8 ms. Despite the straightforward definition of a positive LSR, its intraoperative interpretation is not as simple in real-world scenarios. Various patterns of EMG waves emerge during the monitoring process.

First, the LSR may be undetectable due to patient-specific characteristics or failure in maintaining the anesthetic state. Second, an EMG wave occurring within the 4–8 ms interval is not silent, making the characteristics of the LSR less clear. This phenomenon is a consequence of the spread of the CMAP to the muscle where the recording electrodes are inserted, originating from other facial muscles through interconnections between distant facial nerve branches. Third, inaccurate placement of the recording electrodes may result in the delayed onset latency of the CMAP, making it difficult to properly interpret the EMG findings when a mixture of CMAP and LSR is present. Fourth, there are instances wherein ambiguous findings arise, making interpretations very challenging. Finally, there is a concern that patients who have received multiple botulinum neurotoxin injections may not exhibit accurate EMG findings [54].

## 9. Confirmation of LSR Disappearance

**Figure 3:**
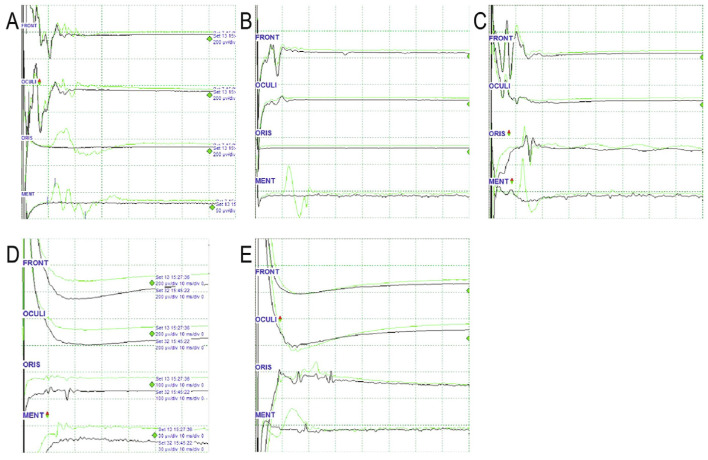
Various patterns of EMG waves in the oris muscle. From the top, each wave represents EMG waves of the frontalis (FRONT), orbicularis oculi (OCULI), oris (ORIS), and mentalis (MENT). Initial (green) and final (black) waves are displayed. The horizontal axis of the grid represents 10 ms, and the vertical axis represents amplitudes of various values. (**A**). Disappearance of LSR: Latency in both the oris and the mentalis is approximately 10 ms. The amplitudes of both waves are similar. (**B**) Undetected LSR: No LSR in the oris is recorded throughout the operation, whereas the characteristic LSR in the mentalis with a latency of 10 ms disappears. (**C**). Persistent LSR: Latencies in both the oris and mentalis are approximately 10 ms. The amplitudes of both waves are similar. The LSR in the oris remains unchanged. (**D**). Spread of CMAP to oris: Latency is shorter, and the amplitude is smaller in the oris than in the mentalis. (**E**). Ambiguous case to interpret: The latency of the waveform in the oris is <10 ms. The amplitude decreases after the surgical procedure. In this case, it is not easy to distinguish between residual LSR and direct oris contraction. EMG, electromyography; LSR; lateral spread response; oris, orbicularis oris. Adapted with permission from copyright holder, Lateral spread response of different facial muscles during microvascular decompression in hemifacial spasm, Clinical Neurophysiology, 132, 2503–2509, 2021 [53].

Confirming the disappearance of the LSR is not as straightforward as confirming its presence (Figure 3). Several factors contribute to the complexity of confirming LSR disappearance. Firstly, there are cases where the LSR is not initially observed at the beginning of the surgery. In most cases, the LSR becomes apparent when the depth of anesthesia is adequately controlled during the procedure. Secondly, the amplitude of the LSR may not completely disappear but rather decrease. In such cases, the presence of additional culprit vessels or incomplete detachment of the culprit vessel from the facial nerve should be considered. Thirdly, the baseline EMG activity may not be completely silent, and spikes near the 10 ms interval can sometimes mask the absence of the LSR. Therefore, any haziness in the baseline EMG at the time of IONM installation should be corrected to ensure efficient monitoring. Lastly, similar to the challenge of confirming a positive LSR, the CMAP from adjacent muscles can sometimes mimic the LSR, making it difficult to detect changes in the LSR.

## 10. Blink Reflex

The blink reflex provides information about the interconnection between the trigeminal and facial nerves. It represents the electrical correlate of the evoked corneal reflex, which is a true reflex involving a sensory afferent limb, intervening synapses, and a motor efferent limb. The afferent limb of the blink reflex is mediated by the sensory fibers of the supraorbital branch of the ophthalmic division of the trigeminal nerve, while the efferent limb is mediated by the motor fibers of the facial nerve. Unilateral stimulation of the supraorbital branch of the trigeminal nerve elicits a bilateral response of the facial nerve, resulting in the blinking of both eyes. The impulses from the supraorbital nerve reach both the main sensory nucleus (at the mid-pons) and the nucleus of the spinal tract (at the lower pons and medulla) of the trigeminal nerve before reaching the bilateral facial nucleus.

The blink reflex consists of two components: an early R1 and a late R2 response. The R1 response is observed only on the side of stimulation, while the R2 response is recorded bilaterally. The R1 response represents the synaptic reflex pathway between the main sensory nucleus of the trigeminal nerve (mid-pons) and the ipsilateral facial nucleus (lower pontine tegmentum). R2 responses are mediated by a more complex pathway between the nucleus of the spinal tract of the trigeminal nerve in the ipsilateral pons and medulla.

In addition to the LSR, monitoring of the blink reflex has the potential to provide an indicator of sufficient decompression, although limited evidence supports its use. The R1 component of the blink reflex, recorded on the ipsilateral side of the symptoms, may disappear after proper decompression of the facial nerve.

## 11. Other Facial Nerve Monitoring Modalities

Free-running EMG is employed to prevent treatment-related complications and provides valuable information about mechanical or thermal injury to the facial nerve. A study by Romstock et al. classified sustained periodic EMG activity lasting several seconds, referred to as the “train”, in free-running EMG [55]. They proposed that the A-train, characterized by a sinusoidal shape with a maximal amplitude of 100–200 microvolts and a frequency of up to 210 Hz, is associated with postoperative facial palsy during cerebellopontine angle tumor surgery, with sensitivity of 86% and specificity of 89%. However, there is currently a lack of systematic evaluation regarding the correlation between free-running EMG activity and postoperative facial palsy or clinical outcomes.

Facial motor evoked potentials elicited through transcranial electrical stimulation are utilized to assess the integrity of the facial nerve. However, there is insufficient evidence to establish the value of the facial motor evoked potential as an indicator of good clinical outcomes in MVD [56,57,58].

F-waves have been reported to be useful during MVD for HFS. F-waves represent the backfiring of the facial motor neurons following antidromic activation. Electrophysiological studies have shown that F-wave appearance is more persistent in patients with HFS and tends to decrease after effective decompression. However, unlike the LSR, F-waves are not specific findings for HFS.

The Z-L response is a facial muscle response evoked by electrical stimulation of the wall of the culprit vessel at the compression site. It promptly disappears after successful decompression. The Z-L response can be a useful alternative when the LSR is not available [59,60,61].

## 12. Recent Studies Discussing IONM of the Facial Nerve during MVD for HFS

The use of neuromuscular blockade (NMB) in surgeries requiring IONM has been a matter of concern. Anesthesiologists argue that not using NMB makes it difficult to control the anesthesia depth and may result in patient movement. However, surgeons believe that NMB can interfere with the smooth functioning of IONM. Consequently, numerous studies have been published on the appropriate use of NMB in patients undergoing IONM. One study concluded that specific LSR monitoring could still be performed with a small amount of NMB [62]. Another study focused on anesthesia, comparing gas anesthesia and total intravenous anesthesia and suggesting that LSR is caused by a central mechanism [63]. As most gas anesthetics act as central nervous system depressants, they can interfere with IONM, making total intravenous anesthesia the preferred choice in neurosurgery requiring IONM. This study divided patients into a gas anesthesia group using desflurane and a total intravenous anesthesia group, and it reported a decrease in LSR amplitude during gas anesthesia, supporting the argument that the LSR is affected by a central mechanism.

In contrast to Wilkinson’s study, recent research by Kameyama suggests a peripheral mechanism for HFS [64]. According to Kameyama, the LSR results from antidromic motor impulses rather than trigeminal sensory input. They propose that slow-conducting motor fibers traveling at 33 m/s give rise to lateral spread, inducing orthodromic impulses. Another study by Wilkinson used facial motor evoked potentials instead of LSR for IONM [56]. The study reported that centrally acting inhaled anesthetic agents suppress facial motor evoked potentials, potentially interfering with IONM. The study concluded that the elevated motor neuron excitability and differential effects of desflurane between the symptomatic and contralateral sides support a mechanism of central pathophysiology in HFS.

Studies have focused on improving the LSR protocol to increase its clinical significance. Hale proposed a method for the detection of the LSR in two muscles, the orbicularis oculi and mentalis [65]. Miao’s study reached a similar conclusion after recording the LSR from multiple muscles [12]. Another study demonstrated that adjusting the stimulation intensity can help in interpreting the LSR [66].

There are also studies that discuss different considerations depending on the surgical findings. It is known that when a facial nerve is compressed by a blood vessel, the nerve becomes indented and discolored. One study claims that the disappearance of the LSR is evident in patients with clear indentations. Patients without clear indentations may not exhibit LSR disappearance, suggesting the presence of another culprit vessel [67]. Another study explored the location of vascular compression based on patients’ symptoms. A patient whose symptoms started at the corner of the mouth showed vascular compression at the cisternal segment of the facial nerve rather than the root exit zone [68].

## 13. Prognostic Value of LSR

Studies investigating the prognostic value of the LSR monitored intraoperatively have yielded inconsistent results [12,13,45,46]. El Damaty conducted a prospective study that reported the usefulness of the LSR in determining the success of decompression during surgery, but the loss of the LSR was not found to be significant for long-term outcomes. Thirumala compared individuals whose LSR disappeared during surgery and those whose LSR did not disappear [69]. The study reported favorable outcomes for those whose LSR disappeared immediately after surgery, but no significant difference was observed between the two groups in the long-term follow-up. Another study suggested that symptoms may persist after surgery if the LSR is not abolished during the procedure [10].

The authors emphasize a particularly significant aspect of this study, which is the impact of how they define “LSR disappearance” on the prognostic value of the intraoperative LSR. They mention that different results were observed depending on the criteria used, such as the partial or complete disappearance of the LSR, or complete or near-total resolution of preoperative symptoms. As a result, it becomes essential to address how these variables can be controlled and standardized to ensure consistency and accuracy in the assessment of treatment outcomes.

To avoid potential biases and discrepancies in the results, the researchers may need to establish clear and standardized criteria for the definition of LSR disappearance. A thorough discussion of this matter is warranted to reach a consensus within the medical community and to facilitate comparability between different studies. By addressing this issue, future research on MVD for HFS can enhance the reliability and clinical significance of its findings, leading to more reliable prognostic information for patients and improved decision making by surgeons.

Despite the use of more sophisticated analytical methods, recent studies have been published indicating that the LSR does not accurately reflect long-term outcomes [70]. Furthermore, another study concluded that IONM of the LSR does not contribute to the prediction of long-term prognosis or immediate postoperative results [12]. However, the inconsistent IONM protocols across multiple studies may explain the discrepancies in their findings.

Recently, studies have been published suggesting that long-term outcomes can be predicted by measuring the LSR in the postoperative period rather than IONM [47]. Among patients with intraoperative LSR loss, 13.8% showed LSR reappearance on postoperative day 2, while 51.7% of patients without intraoperative LSR loss showed LSR disappearance on the same day. This is the first study to highlight the clinical utility of postoperative LSR measurement. Another study was published demonstrating the usefulness of the LSR assessed one month after surgery as a long-term prognostic factor [42]. LSR measurements were performed one month postoperatively on 883 patients who underwent MVD for HFS. In this study, a persistent LSR at 1 month postoperatively predicted a poor outcome one year after the procedure. When residual symptoms and a persistent LSR were simultaneously observed at one month postoperatively, the probability of experiencing residual symptoms one year after surgery was predicted to be as high as 50.7%. Hence, early revision surgery could be considered in such cases. This study reveals that postoperative LSR measurement can assist in predicting outcomes and determining the optimal timing for revision surgery.

## 14. Conclusions

IONM has evolved over time, and its application has expanded to various surgical fields. However, challenges such as the need for specialized personnel, potential inaccuracies due to fatigue or loss of concentration, and the integration of clinical information during surgery remain. Further advancements in IONM technology and collaboration between surgeons and anesthesiologists can address these challenges and improve the monitoring process.

In MVD surgery for HFS, the lateral spread response (LSR) is a valuable indicator in confirming the successful decompression of the facial nerve. Recent modifications to the protocol, such as the inversion of the stimulating electrode position, have shown potential in enhancing the efficacy of LSR monitoring. In addition to the LSR, the blink reflex provides important information about the interconnection between the trigeminal and facial nerves. It helps to assess the integrity of these nerves and their pathways, further contributing to the success of MVD surgery for HFS. The article mentions other facial nerve monitoring modalities, such as free-running EMG, facial motor evoked potentials, and F-waves, but their clinical significance and value in MVD for HFS are not well established. Recent studies exploring the LSR have also been summarized.

In summary, IONM is a valuable tool in MVD surgery for HFS, facilitating the identification and decompression of the culprit vessel, monitoring critical nerve function, and improving surgical outcomes. Continued research and technological advancements will further enhance the role of IONM in neurosurgical procedures, benefiting patients with HFS and other related disorders.

## Data Availability

Data is contained within the article.
